# Progress in Biological Research and Treatment of Pseudomyxoma Peritonei

**DOI:** 10.3390/cancers16071406

**Published:** 2024-04-03

**Authors:** Xi Li, Guodong Liu, Wei Wu

**Affiliations:** 1 Department of Geriatric Surgery, Xiangya Hospital, Central South University, Changsha 410008, China; xilixy@csu.edu.cn; 2 Department of General Surgery, Xiangya Hospital, Central South University, Changsha 410008, China; 3 National Clinical Research Center for Geriatric Disorders, Xiangya Hospital, Central South University, Changsha 410008, China

**Keywords:** pseudomyxoma peritonei, cytoreductive surgery, hyperthermic intraperitoneal chemotherapy, mucin, mucolytic, targeted therapy

## Abstract

**Simple Summary:**

Pseudomyxoma peritonei (PMP) is a rare disease and has, thus, been the focus of relatively few studies in the field of digestive system research. Even experts and scholars in this field have certain deficiencies in their understanding of the disease. Although the standard treatment of cytoreductive surgery (CRS) combined with hyperthermic intraperitoneal chemotherapy (HIPEC) has improved patient prognosis, problems such as the difficulty of operation, tumor recurrence, single treatment method, and poor quality of life cannot be properly solved. This review mainly examines the progress of biological research and the existing or potential treatment strategies in relation to pseudomyxoma peritonei. It is expected to help scholars in related fields to understand the disease and provide potential directions for research into more effective and personalized treatment strategies.

**Abstract:**

Pseudomyxoma peritonei (PMP) is a rare disease characterized by extensive peritoneal implantation and mass secretion of mucus after primary mucinous tumors of the appendix or other organ ruptures. Cytoreductive surgery (CRS) combined with hyperthermic intraperitoneal chemotherapy (HIPEC) is currently the preferred treatment, with excellent efficacy and safety, and is associated with breakthrough progress in long-term disease control and prolonged survival. However, the high recurrence rate of PMP is the key challenge in its treatment, which limits the clinical application of multiple rounds of CRS-HIPEC and does not benefit from conventional systemic chemotherapy. Therefore, the development of alternative therapies for patients with refractory or relapsing PMP is critical. The literature related to PMP research progress and treatment was searched in the Web of Science, PubMed, and Google Scholar databases, and a literature review was conducted. The overview of the biological research, treatment status, potential therapeutic strategies, current research limitations, and future directions associated with PMP are presented, focuses on CRS-HIPEC therapy and alternative or combination therapy strategies, and emphasizes the clinical transformation prospects of potential therapeutic strategies such as mucolytic agents and targeted therapy. It provides a theoretical reference for the treatment of PMP and the main directions for future research.

## 1. Introduction

**Figure 1:**
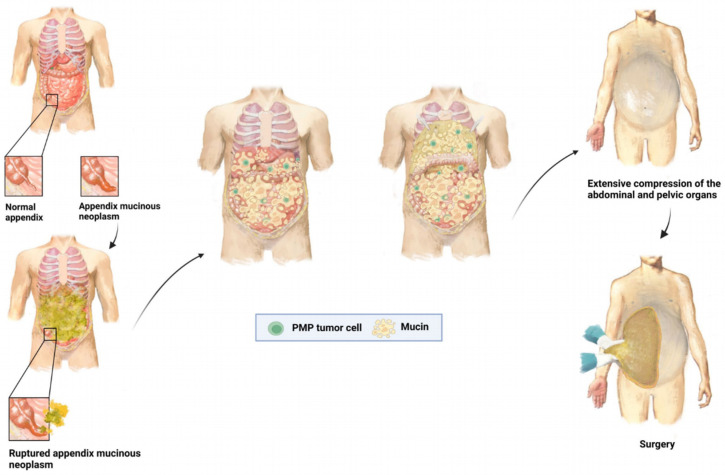
Schematic model of the pathogenesis and development of pseudomyxoma peritonei.

**Figure 2:**
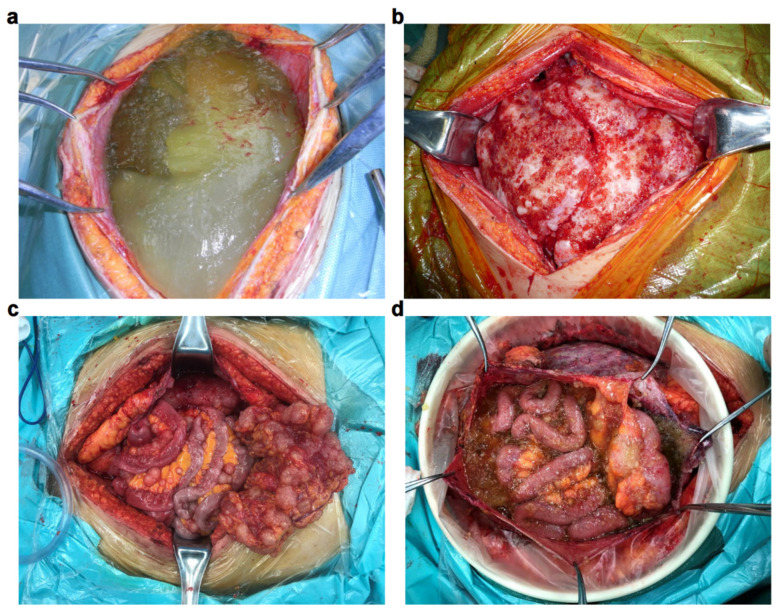
Intraoperative representations of different tumor growth types: (**a**) Jelly type, abdominal and pelvic lesions are dominated by a large amount of jelly-like mucus. (**b**) Plate type, the lesions are fused into a plate shape, with a hard texture, and invade multiple organs in the abdominal and pelvic cavity. (**c**) Nodular type, a large number of tumor nodules diffusely distributed in the abdomen and pelvis, with different sizes, most of which are in the omentum, mesentery, and intestinal serosa. (**d**) Mixed type, the lesions present a mixture of various types and various manifestations.

Pseudomyxoma peritonei (PMP) is an internationally recognized rare disease with an incidence of 2–4 per million [1,2,3]; the incidence is higher in females, approximately 2–3 times that of males [4,5,6]. It is often caused by the rupture of a mucinous tumor originating from the appendix, followed by massive colonization of tumor cells in the peritoneal cavity and continued production of mucus. Cases of PMP caused by mucinous tumors originating from organs such as the ovaries, colon, pancreas, and urachus have also been reported [7,8,9,10] (Figure 1). Although the manifestations of intra-abdominal mucinous tumor tissue may vary among different patients (Figure 2), under the premise of non-surgical intervention, such patients will suffer from progressive intestinal obstruction and nutritional deficiencies caused by progressive mucus compression, eventually developing cachexia, which leads to death.

The prognosis of PMP is highly correlated with pathological classification [11]. However, over the years, there has been much controversy over the pathological grading method for PMP, which makes it difficult to standardize and unify pathological reports and to reference and combine data from disparate diagnosis and treatment centers. Various grading methods have been reported in the past [1,12,13,14,15,16,17], and Table 1 shows three commonly used clinical pathological types. At present, the four-level classification system proposed by the Peritoneal Surface Oncology Group International (PSOGI) in 2016 [1] has been widely recognized and accepted by front-line clinical workers around the world. 

In 1980, Spratt et al. [18] first applied cytoreductive surgery (CRS) combined with hyperthermic intraperitoneal chemotherapy (HIPEC) in the clinical treatment of PMP patients, and since then, the prognosis of PMP patients has significantly improved [19]. According to the latest large sample retrospective analysis by Kusamura et al. [20], the 5-year survival rate of 1548 PMP patients who received CRS-HIPEC reached 57.8%. CRS-HIPEC has become the standard and preferred protocol for PMP treatment and represents a historical milestone in PMP treatment [21,22]. Although the overall survival time of PMP patients has been significantly prolonged, relevant studies have reported that approximately 24.2% of patients have tumor recurrence and progression after CRS-HIPEC [23]. Although the rate of invasion of PMP is significantly lower than that of other malignant tumors, and despite the fact that most patients can survive with tumors for a long time, when intestinal obstruction occurs again or other conditions caused by tumor recurrence require hospitalization for treatment, the therapeutic effect of CRS-HIPEC is often poor, and some patients lose the opportunity to repeat CRS-HIPEC. For these patients and for those who cannot receive standard treatment after assessment at the first visit, the current treatment methods are limited [24,25,26,27,28]. Therefore, it is necessary to explore new and effective treatment strategies.

The main purpose of this review is to provide clinicians with more standardized and diverse treatment methods by summarizing the current basic research progress, treatment status, potential treatment strategies, research limitations, and future research directions of pseudomyxoma peritonei. At the same time, it provides researchers in this field with potentially feasible clinical translation research directions and draws attention to the limitations of existing research on this disease.

## 2. Materials and Methods

The literature search was conducted in Web of Science, PubMed, and Google Scholar databases, using individual and combined keywords: “pseudomyxoma peritonei”, “peritoneal mucinous carcinomatosis”, “peritoneal adenomucinosis”, “mucinous tumors”, “appendiceal mucinous neoplasms”, “treatment”, “therapy”, “cytoreductive surgery”, “hyperthermic intraperitoneal chemotherapy”, “intraoperative intraperitoneal chemotherapy”, “prognosis”, “mucin”, “mucus”, “biology”, “sequencing”, “molecular”, “mutation”, “ profiling”, and “gene expression”. Through a preliminary screening of paper titles and abstracts, full texts that match the topic were retrieved, and a detailed manual review was conducted to further confirm the quality of the research and the relevance of the topic. 

## 3. Progress in Biological Research 

### 3.1. Overview of Common Molecular Mutations 

The mutation rate of Kirsten rat sarcoma viral oncogene homolog (KRAS) in PMP is approximately 77.8% [29,30,31,32,33,34,35,36,37]. The most common mutation site is located at G12D and G12V [30]. The mutation of the KRAS gene may promote the proliferation of mucous-producing tumor cells and the secretion of mucin (MUC) by activating the mitogen-activated protein kinase (MAPK) downstream signaling pathway [29,38,39]. In addition, Pietrantonio et al. [34] found that KRAS mutations were significantly associated with lower progression-free survival (PFS) in PMP patients. However, KRAS status is not an independent factor affecting overall survival (OS) and seems to have no significant influence on OS [36,40]. 

The mutation rate of guanine nucleotide-binding protein alpha subunit (GNAS) is approximately 45.7% [31,32,33,35,37,41]. The most common mutation sites of GNAS in PMP are located at R201C and R201H [41]. Nishikawa et al. [42] found that GNAS mutation could increase the expression levels of MUC2, MUC5AC, and cyclic adenosine monophosphate (cAMP) in colon cancer cells, and this process could be reversed by Protein Kinase A (PKA) inhibitors. This suggests that GNAS mutations may be involved in regulating mucin expression through the cAMP-PKA signaling pathway. However, this conclusion needs to be verified in PMP cell lines. Additionally, relevant studies have reported that GNAS mutation is not significantly correlated with the prognosis of patients [34,36]. 

Importantly, a large number of studies have shown that mutations in KRAS and GNAS often occur simultaneously in patients with PMP [30,33,34,43,44,45,46,47]. This indicates that there may be an interaction between KRAS and GNAS which needs to be further discussed in subsequent basic research.

The overall mutation rate of TP53 is approximately 16.3% [30,31,32,33,34,41,46,48,49,50]. TP53 is closely related to the pathological classification of PMP, and the mutation rate is often higher in peritoneal mucinous carcinomatosis (PMCA) patients [30,31,32,44]. Noguchi et al. [31] found that TP53 mutation was also associated with malignant characteristics of PMP. In addition, relevant studies have shown that PMP patients with TP53 mutations tend to have worse PFS and OS [32,40,51]. 

Other common mutated genes in PMP patients include SMAD family member 4 (SMAD4) (15.7%) [30,32,33,41,48,52], APC regulator of the WNT signaling pathway (APC) (10.4%) [30,33,41,48,52], and phosphatidylinositol-4,5-bisphosphate 3-kinase catalytic subunit alpha (PIK3CA) (5.9%) [30,31,32,41,48]. In addition, Yan et al. [53] found that approximately 6.3% of PMP patients had mismatch repair (MMR) gene mutations, which were associated with poor prognosis.

### 3.2. Molecular Subtypes 

Some researchers have noted that the gene expression of PMP patients can be divided into immune-enriched, mixed, and oncogene-enriched types [54,55]. The immune-enriched type mainly involves the increased expression of genes related to immune cell function, such as the natural killer cell-related gene killer cell lectin-like receptor F1 (KLRF1) and killer cell lectin-like receptor G1 (KLRG1) and the T-cell-related genes’ T-cell receptor alpha locus (TRA) and T-cell receptor beta constant 1 (TRBC1). The oncogene-enriched type mainly involves upregulated genes related to tumor cell proliferation and invasion, including Claudin3 (CLDN3), Claudin4, serine peptidase inhibitor Kazal type 1 (SPINK1), epithelial splicing regulatory protein 1 (ESRP1), and epithelial cell adhesion molecule (EpCAM) [54,55]. In the mixed type, the gene expression of both subtypes is partially elevated. The results show that the molecular subtype is an independent prognostic factor. Patients with the oncogene-enriched type have the shortest median survival (1.4 years), those with the immune-enriched type have the longest median survival (7.7 years), and those with the mixed type fall between the two (3.6 years) [54]. The molecular subtype is expected to play an important role in treatment selection and the preliminary judgment of patient prognosis after being verified in a large number of clinical patients in the future.

### 3.3. Mucin 

Mucin (MUC) is a highly glycosylated protein named MUC 1–20, according to the sequence of its discovery. Its main physiological role is to lubricate catheters and body cavities and to act as a chemical barrier to isolate harmful substances [56,57]. Mucins are mainly divided into transmembrane mucins (MUC1, MUC3, MUC4, MUC12, MUC13, MUC15, MUC16, MUC17, MUC21, and MUC22) and secreted mucins, which, in turn, can be divided into gel-forming mucins (MUC2, MUC5AC, MUC5B, and MUC6) and monomeric mucins (MUC7 and MUC20) [58,59,60]. In PMP, secreted gel-forming mucins are the main component of mucus, and its mass secretion and character change are considered to be one of the most important pathological processes, as well as the main reasons leading to the clinical symptoms and poor prognosis of patients [30,53,61]. The four secreted gel-forming mucin genes are located on the p15 arm of chromosome 11 and are regulated by a variety of factors at the transcriptional level, such as hormones, bacterial products, retinoic acid, growth factors, transcription factors activating transcription factor 1 (ATF1), retinoic acid receptor alpha (RAR-α), cAMP responsive element-binding protein (CREB), proinflammatory factors interleukin 1 beta (IL-1β), tumor necrosis factor alpha (TNF-α), and pleiotropic cytokines IL-9 and IL-13 [62]. The microscopic structure of the protein is rod-shaped, mainly composed of the central PTS sequence (Pro-Thr-Ser sequence repeated at high frequency) and the polysaccharides on the outer edge. The PTS sequence is modified by O-glycosylation (above 80%) and N-glycosylation to form the core domain of mucin, and it forms dimers or multimers by establishing disulfide bonds between the domains [60,63]. MUC2 and MUC5AC are the most highly expressed mucins in PMP patients, with positive rates of 99.1% and 96.5%, respectively [41]. MUC2 is the most abundant component in PMP tumor tissue and is often wrapped around tumor cells to form a barrier, greatly limiting the effect of intravenous or abdominal infusion chemotherapy drugs and greatly increasing the difficulty of tumor reduction surgery, which is a key problem to be solved in basic and preclinical studies [64]. Mucus sclerosis is also a key pathological mechanism of PMP disease progression [61,63]. Pillai et al. [65] found that MUC5AC and MUC5B play an important role in the hardening of mucus, and the different ratios of the two to MUC2 may determine the characteristics of the mucus in the abdominal cavity (soft mucus, semihard mucus, and hard mucus). In addition, Mall et al. [66] found the presence of the cross-model mucin MUC4 in PMP patients, but its function was not further elaborated. Some scholars speculated that MUC4 might also affect the mucus soil of PMP [65]. At present, there are relatively few studies on MUC6, and further studies are needed to explore its function.

### 3.4. Epithelial–Mesenchymal Transition (EMT) 

EMT not only plays a key role in organ development, fibrosis, and tissue healing but also participates in the process of tumor occurrence and development, including growth invasion, distant metastasis, inhibition of apoptosis, immune escape, improvement of resistance to radiotherapy and chemotherapy, and interaction with the immune microenvironment [67,68,69,70]. A large number of studies have shown that EMT is mainly regulated by EMT transcription factors [67,71]. They inhibit the expression of the epithelial structural gene cadherin 1 (CDH1) and activate the mesenchymal phenotype-related genes fibronectin 1 (FN1), CDH2, and vimentin (VIM) by binding to the promoter sequences of related genes [72,73,74,75,76]. EMT may also play an important role in PMP. It has been reported that the expression of N-cadherin is increased and that of E-cadherin is decreased in PMP tumor cells [77]. This suggests that PMP tumor cells may mainly exhibit a mesenchymal phenotype, which makes the loss of polarity of tumor cells more likely to spread in the abdomen and pelvis, consistent with the characteristics of widespread cultivation of PMP. Other studies compared the expression of mesenchymal markers between single tumor cells and tumor cell clusters in PMP and found that single tumor cells had more obvious EMT status, E-cadherin negativity, and strong vimentin positivity, while cell clusters exhibited contrary results [16,78]. This is consistent with clinical results. More single tumor cells are often seen in PMCA than in disseminated peritoneal adenomucinosis (DPAM), and the more single cells there are, the worse the prognosis [78]. In addition, SMAD4 is frequently mutated in PMP patients, with a mutation rate of approximately 15.7% [41]. Xiong et al. [79] found that SMAD4 could regulate the expression of zinc finger E-Box binding homeobox factors (ZEB1) by activating the signal transducer and activator of the transcription 3 (STAT3) signaling pathway in colorectal cancer and thus participate in the EMT process. This process may also exist in PMP, but it needs to be confirmed by relevant studies. 

### 3.5. Intestinal Flora 

Microorganisms have been proven to influence the disease progression of a variety of tumors [80,81,82]. As mentioned above, PMP is mainly formed after the collapse of appendix mucus tumors, during which various bacteria originally existing in the appendix enter the abdomen and pelvic cavity. Semino-Mora et al. [83] were the first to discover the presence of *Helicobacter pylori* (HP) in PMP-excised specimens and observed that the bacterial density and MUC2 expression level of PMCA were significantly higher than those of DPAM, and the bacterial density was correlated with the MUC2 expression level. On this basis, Gilbreath et al. [84] further explored the classification of bacteria in tumor tissue and mucus samples of PMP patients and found that the core microbiome of PMP patients was mainly composed of four phyla, *Proteobacteria* (77%), *Actinobacteria* (15%), *Firmicutes* (5.7%), and *Bacteroidetes* (2.3%), and the bacterial communities in tumor tissue and mucus samples were highly similar. These microorganisms were found to be able to adhere to secreted MUC2 in vitro. Lo et al. [85] found a new bacterial species named PMP191F in PMP samples, and it was also able to bind to MUC2. However, the interaction relationship and mechanism between these bacteria and MUC2 have not been reported. Villarejo-Campos et al. [86] performed 16S sequencing on PMP mucinous tumor tissue, and the results suggested that *Proteobacteria* was the dominant phylum and *Pseudomonas* was the dominant genus. Previous studies have shown that *Pseudomonas* is directly related to the overexpression of mucins [87]. In addition, Semino-Mora et al. [88] conducted a clinical trial to observe the effect of antibiotics on PMP bacterial density and β-catenin, and they found that the bacterial density was significantly reduced, and the β-catenin expression level was significantly increased after antibiotic treatment. β-catenin, E-cadherin, and actinin-4 are involved in intercellular adhesion and movement and are important molecules in maintaining the polarity of epithelial cells [89]. Bacteria may induce EMT by inhibiting β-catenin expression levels so that the connection mechanism between PMP tumor cells is damaged, thus losing cell polarity and spreading in the abdomen and pelvic cavity. However, direct research is needed to confirm this hypothesis.

## 4. Treatment 

### 4.1. CRS-HIPEC

Since Spratt et al. [18] first proposed the combined application of CRS and HIPEC in the treatment of PMP in 1980, a large number of studies have shown that CRS-HIPEC is effective and safe for treating this disease [19,20]. The 5-year and 10-year survival rates for PMP patients treated with conventional debulking surgery are only 15.3–67% and 8.3–31%, respectively [90,91]. However, Chua et al. [19] reviewed the data of 2298 patients with PMP who underwent CRS-HIPEC at multiple treatment centers and found that the 10-year and 15-year survival rates were as high as 63% and 59%, respectively, and the median disease-free survival (DFS) and OS were 8.2 and 16.3 years, respectively. These findings indicate that CRS-HIPEC, as a therapeutic measure, can significantly improve the prognosis of patients with PMP. Therefore, the PSOGI has also issued a number of guidelines recommending CRS-HIPEC as the standard treatment for PMP patients, and these guidelines are currently widely recognized [92]. In addition, studies have indicated that the number of PMP patients admitted to diagnosis and treatment centers each year directly affects the therapeutic effect of CRS-HIPEC, and fewer than 60 cases of PMP are independent predictors of treatment failure [93,94]. Therefore, it is recommended that patients with PMP be concentrated in treatment centers with rich practical experience.

The cornerstone of PMP treatment is carrying out CRS to remove the tumor lesions visible to the naked eye as much as possible. With the development of surgical techniques and the accumulation of experience, the current CRS strategy is characterized by a set of standardized procedures [13,95]. First, after opening the peritoneum, a comprehensive exploration of the abdominal and pelvic cavity is needed, and the peritoneal cancer index (PCI) score is determined [96]. Then, the left upper peritoneum, right upper peritoneum, parietal anterior peritoneum, greater omentum, lesser omentum, spleen, and pelvic peritoneum are excised, and the stomach, small intestine, colon, and other widely implanted organs are excised as appropriate according to the individual situation of the patient. Finally, the completeness of the cytoreduction (CC) score is determined after surgery, according to the degree of CRS [97]. CRS reaching CC0 or CC1 is called complete cytoreduction surgery (CCRS). The reduction degree of tumor lesions is significantly related to the prognosis of PMP patients, and even high-grade PMP patients can obtain better OS and DFS after reaching CCRS [19,98]. Therefore, for the vast majority of PMP patients, CCRS should be achieved in surgery as much as possible, but some contraindications should be considered [92] (Table 2).

HIPEC has unique advantages as an effective treatment for PMP patients. (i) Regarding pharmacokinetics, because the peritoneal–plasma barrier restricts the absorption of macromolecular chemotherapeutic drugs into the blood, intraperitoneal administration can often involve maintaining a high concentration of local drugs in the abdomen, while keeping the systemic drug level low. Furthermore, the concentration of intraperitoneal administration can be 1000 times higher than that of intravenous administration [99,100]. (ii) In terms of the thermal effect, a large number of studies have shown that, in the range of 41~43 °C, the thermal effect has multiple inhibitory effects on tumor cells, while normal tissue cells are less affected [101,102]. This is related to an imbalance of the autostabilization mechanism caused by the increase in lysosome number and lysosomal enzyme activity in tumor cells, as well as the insufficient nutrient supply caused by reduced or even complete interruption of blood flow [103,104]. (iii) Regarding the synergistic effect, additive interaction exists between the thermal effect and the cytotoxicity of drugs, as has been confirmed in multiple studies [102,105]. This may be related to the increase in membrane permeability and the change in drug pharmacokinetics due to the thermal effect [106]. However, HIPEC also has an obvious disadvantage: insufficient penetration depth (3–5 mm) [107,108]. In PMP patients, the tumor load in the abdominal cavity is large. If the residual tumor tissue is not controlled within the penetration range of HIPEC by CRS, it will be difficult to effectively kill tumor cells. This is also the reason why 2.5 mm is used as the threshold to distinguish CCRS. Only when CCRS is achieved can HIPEC be used to obtain the best effect. 

Currently, HIPEC protocols used to treat PMP are based mainly on oxaliplatin or mitomycin C [92,109,110,111]. Due to the lack of sufficient prospective evidence, there has been controversy regarding the use of the program, and no international consensus has been formed. To compare the true effects and toxic side effects of the two, Levine et al. [112] conducted a multicenter randomized controlled trial in 2018 in patients with appendiceal-derived PMP. The results showed that there was no significant difference in the incidence of platelets and leukopenia between the oxaliplatin group and mitomycin C group, and the 3-year OS (86.9% vs. 83.7%) and DFS (64.8% vs. 66.8%) were similar. However, the oxaliplatin group reported better emotional and physical well-being. In 2020, PSOGI also launched expert voting for different HIPEC schemes [92], and the results are shown in Table 3. However, none of these approaches reached the expert consensus threshold (>50%). It is expected that more clinical trials will be conducted in the future to reach a consensus on this issue.

### 4.2. Maximum Tumor Debulking (MTD)-HIPEC

Although CCRS-HIPEC is an ideal treatment for PMP, it is not always possible to achieve CCRS based on the individual situation of each patient, especially for patients with relapse, extensive small intestine involvement, and poor underlying conditions. For them, achieving CCRS at all costs may result in poorer quality of life and more serious surgical complications [115]. However, tumor reduction without surgery relying only on relatively insensitive chemotherapy and other measures cannot achieve the effect of alleviating patients’ symptoms [24,25,26,28]. The idea of MTD provides a new choice for such patients. It aims to reduce the tumor load in the abdominal cavity as much as possible, while solving the main symptoms of patients, such as obstruction, without sacrificing the vast majority of abdominal organs and greatly increasing the probability of intestinal fistula and other serious complications in exchange for the complete reduction of tumor cells [92,116]. Delhorme et al. [116] conducted a retrospective study of 39 patients who underwent MTD-HIPEC and found that the median OS and DFS reached significance at 55.5 months and 20 months, respectively. Alves et al. [117] reported that 20 patients who received MTD-HIPEC showed significant improvements in appetite, mood, and health-related quality of life (HRQL) 1 year after surgery. In a vote of experts initiated by PSOGI in 2020, 98.2% of them recommended that, for patients unable to undergo CCRS, MTD should be performed in experienced treatment centers, and 60.7% recommended routine HIPEC after MTD [92]. However, there is still a lack of prospective research evidence to prove that MTD is superior to CCRS for this type of PMP patient, and the criteria for patient selection for MTD need to be further clarified.

### 4.3. Early Postoperative Intraperitoneal Chemotherapy (EPIC)

EPIC is also a common intraperitoneal chemotherapy method. Unlike HIPEC, EPIC does not require heat, but the treatment period is longer. Generally, a cycle of 24 h lasts for 5 days (1–5 days after surgery), and 5-fluorouracil is commonly used for chemotherapy [118]. On the basis of low tumor load after CRS, long-term contact between chemotherapy drugs and abdominal residual lesions would theoretically have greater advantages [13]. Chua et al. [118] retrospectively analyzed the prognosis of 108 patients with low-grade malignant PMP who received CRS-HIPEC/EPIC (*n* = 21) or CRS-HIPEC-EPIC (*n* = 87). The 5-year survival rate was higher in the CRS-HIPEC-EPIC group than in the CRS-HIPEC/EPIC group (86% vs. 64%). Huang et al. [119] found that EPIC combined with CRS-HIPEC was an independent influencing factor for improved prognosis in patients with both low- and high-grade PMP. There was no significant difference with the CRS-HIPEC group in terms of total length of hospital stay, in-hospital mortality, and incidence of serious complications. However, other relevant studies have reached completely different conclusions. The combined use of EPIC has been shown to not only not significantly improve OS and DFS but also increase the incidence of grade III/IV complications [120,121]. Since the above studies were retrospective, prospective clinical trials with large samples are needed to draw conclusions and resolve these contradictory findings. In 2020, PSOGI launched an expert vote on this issue, and 60.7% of the experts supported EPIC after CRS-HIPEC, while 39.3% were against it [92].

### 4.4. Hyperthermic Intrathoracic Chemotherapy (HITHOC)

Invasive changes in the pleura occur in approximately 5.4% of PMP patients and are often associated with poor prognosis [122]. The reason for this phenomenon may be related to the direct invasion of subphrenic lesions, lymphatic vessel metastasis of thoraco-abdominal communication, and implantation caused by accidental injury of the diaphragm during surgery [122,123,124]. The basic process of HITHOC is similar to that of HIPEC, and keeping the diaphragm open during this process enables both HITHOC and HIPEC to be performed simultaneously [125]. Several studies have shown that HITHOC may be a potential treatment for patients with pleural involvement of PMP [122,124,125,126]. However, Ashraf-Kashani et al. [127] found that HITHOC could lead to hemodynamic changes in patients. At present, most of these studies are presented in the form of case reports, the level of evidence is weak, and the safety of this approach needs to be further investigated.

### 4.5. Neoadjuvant Chemotherapy

Given the advantages of neoadjuvant chemotherapy for certain tumors, such as reducing tumor load, increasing the probability of radical resection, and improving prognosis [128,129], some researchers have also discussed related aspects of PMP. The results show that neoadjuvant chemotherapy does not improve the prognosis of patients with both low- and high-grade PMP and can even lead to lower OS and PFS [130,131,132,133]. However, Milovanov et al. [133] found that neoadjuvant therapy improved OS at 1, 2, and 3 years in patients with peritoneal mucinous carcinomatosis with signet ring cells (PMCA-S) (94%, 67%, and 22% vs. 43%, 14%, and 14%). In addition, regarding the use of neoadjuvant chemotherapy, 87.3% of the PSOGI panel recommended fluoropyrimidine and an alkylating agent combination [92]. In conclusion, current evidence suggests that patients pathologically classified as PMCA-S may benefit from neoadjuvant chemotherapy but that this therapeutic strategy is not suitable for the vast majority of PMP patients, which may be related to the mucus barrier around tumor cells.

### 4.6. Systemic Chemotherapy

The need for systemic chemotherapy after CRS-HIPEC is a little-studied clinical topic in PMP. Small-sample retrospective experiments from different centers have reported completely different results. Blackham et al. [131] (*n* = 22) found that, compared with CRS-HIPEC alone, additional systemic chemotherapy improved OS and PFS in patients with high-grade PMP. However, other studies have shown that postoperative systemic chemotherapy has no significant effect on OS or even has a negative effect [132,134]. In addition, the PSOGI expert group recommended that adjuvant chemotherapy should be considered after completion of CRS-HIPEC for PMP patients with a pathological classification of PMCA or PMCA-S (89.1%) and should not be completely abandoned for low-grade PMP (90.9%) [92]. However, it is undeniable that the effectiveness of systemic chemotherapy currently lacks direct medical evidence. In the future, it is necessary to conduct multicenter large-sample clinical studies to draw reliable conclusions to better regulate the treatment of PMP.

### 4.7. Palliative Chemotherapy

Palliative chemotherapy is a potential treatment for PMP patients who relapse or cannot be treated surgically due to their underlying conditions. At present, there is a lack of unified standards for chemotherapy regimens, and most centers use oxaliplatin- and 5-fluorouracil-based chemotherapy regimens [24,25,26,28]. Farquharson et al. [24] conducted a phase II trial and found that 15 out of 40 patients with unresectable PMP achieved significant clinical and radiographic responses after palliative chemotherapy (mitomycin C and capecitabine). Two patients were followed up with CRS-HIPEC due to good results. The overall 1- and 2-year OS rates were 84% and 61%, respectively. A series of subsequent experiments found that the disease control rate (DCR) after palliative chemotherapy was 65–88%, the median PFS was 8–13 months, and the median OS was 26.2–27.9 months [25,26,28]. The chemotherapies used included FOLFOX4 [26], mFOLFOX6 [25], and capecitabine combined with cyclophosphamide [28]. In addition, other studies found that the combination of bevacizumab, a monoclonal antibody targeting vascular endothelial growth factor (VEGF), can significantly improve PFS and OS in patients with PMP, especially for patients with high-grade PMP [46,135]. Therefore, more than 90.9% of the PSOGI expert group recommended that palliative chemotherapy be considered for patients with PMP who were assessed as unfit for surgery or unresectable, regardless of the pathologic type of PMP, and 78.2% recommended the combination of a single angiogenic inhibitor (such as bevacizumab) [92]. 

### 4.8. Pressurized Intraperitoneal Aerosol Chemotherapy (PIPAC)

While HIPEC has many advantages, it also has defects such as uneven distribution of drugs and poor permeability in the abdominal cavity [107,108,136]. To address these challenges, Solaßs et al. [136] reported on PIPAC, a new intraperitoneal chemotherapy technique. The basic principle of this technology is to press the abdominal cavity through an operation mode similar to laparoscopy and combine it with “atomized” chemotherapy drugs so that it can better diffuse to the entire abdominal cavity and improve the permeability to play a killing role [137,138]. Relevant studies have shown that PIPAC applied to patients with peritoneal metastasis of gastrointestinal, appendix, ovary, and other malignant tumors can cause regression of peritoneal nodules or fibrosis, with good tumor response and safety [137,139,140,141,142]. Only one clinical application of PIPAC in patients with PMP has been reported. After three cycles of PIPAC (cisplatin 7.5 mg/m^2^ combined with doxorubicin 1.5 mg/m^2^, 12 mmHg, 37 °C, 30 min), the patient’s abdominal mucus and ascites basically disappeared, and 66% of intraoperative biopsy tissues indicated fibrosis and inflammation, with no tumor cells found [143]. However, the actual efficacy and safety of PIPAC in PMP patients remain to be evaluated. In addition, the comparison of efficacy between PIPAC and HIPEC is also a worthy clinical topic.

## 5. Potential Treatment Strategies

### 5.1. Mucolytic Therapy

The mass secretion, accumulation, and fibrous sclerosis of mucin in the abdomen and pelvic cavity are very difficult situations for surgeons to face during an operation. For patients with extensive abdominal and pelvic organ involvement, it is difficult to choose among the scope of surgical resection, the degree of tumor reduction, the incidence of serious complications, and postoperative quality of life. Under ideal conditions, the degradation of the accumulated mucus in the body through the use of a dissolving agent and its extraction outside the body can not only relieve the compression symptoms of the patient’s abdomen nonoperatively but also improve the possibility of CCRS during the operation, avoid the occurrence of multisite combined organ resection and serious surgical complications, and improve the therapeutic effect of HIPEC on the residual tumor cells in the abdominal cavity.

Mucin is the main component of intraperitoneal mucus in PMP and is the core solution target of dissolving agents in mucolytic therapy. Glycosidic bonds and disulfide bonds are the main chemical bonds for mucin polymerization and function [60]. Therefore, any chemical that acts on either the glycosidic or disulfide bonds could theoretically degrade mucin. In the past, relevant scholars have tried to use sodium bicarbonate and glucose to dissolve mucus in PMP patients, but these reagents have mediocre effects and certain side effects, and they have not been further studied or promoted in clinical practice [144,145,146]. In recent years, the use of a combination reagent of bromelain and N-acetylcysteine (NAC) developed by the Morris team has achieved significant mucolytic effects and high safety in preclinical studies and clinical trials. This represents a promising development for the clinical transformation and application of mucolytic therapy [147,148,149,150]. 

Bromelain is a mixed enzyme extracted from the flesh, root, and skin of pineapple that can effectively hydrolyze glycosidic bonds [151]. NAC is a kind of respiratory suppurative mucolytic agent that has been widely used clinically and mainly relies on the destruction of disulfide bonds to complete the decomposition of mucin [152,153]. Morris’s team found, in the initial stage of the experiment, that 300 mg/mL bromelain +4% NAC could dissolve mucus at 3 h in vitro and 72 h in vivo without significant toxic side effects. In addition, they found that the combined reagents had similar mucolytic effects at 37 °C and 41 °C, suggesting that the combined reagents were effective even during HIPEC [149]. On this basis, they also found that the combination reagent effectively reduced the expression and secretion of MUC2 and MUC5AC both in vitro (using LS174T colon cancer cells that expressed specific mucins instead of PMP cells) and in vivo [147]. They then verified its safety in a rat model, and the results showed that intraperitoneal injection of the combined agent was safe and had no significant impact on the healing of the colonic anastomosis. However, this study used a healthy rat model, and the impact on animal models under disease conditions is still unknown [154]. Following these positive preclinical findings, the team conducted a multicenter prospective phase II trial to investigate the clinical efficacy of bromelain in combination with NAC in patients with recurrent peritoneal mucinous tumors and PMP (NCT 03976973). The preliminary results showed that, in 20 patients with peritoneal disease (including 6 with PMP), 73.2% of the treatment sites showed an objective response to extracting gelatinous or liquid tumor tissue directly from the drainage tube. In addition, complications were controllable [150]. However, this study had a small sample size and lacked long-term prognostic results. In the future, more patients need to be included, and long-term prognostic results need to be tracked to support the conclusions of this study.

In addition, the team found that the combination of reagents significantly inhibited the proliferation of gastric and colon cancer cells (including the LS174T cell line) through synergistic and additive interactions. Its role may be related to interfering with the growth cycle of tumor cells and inducing apoptosis and autophagy [148]. However, whether it can exert the same inhibitory effect in real PMP cell lines remains to be further verified.

### 5.2. Antiangiogenic Therapy

Due to the small number of tumor cells and the large amount of mucus associated with PMP, ordinary conventional chemotherapy drugs cannot play an effective role. Relevant studies have shown that antiangiogenic therapy may be an effective treatment [155,156]. Dohan et al. [156] confirmed the existence of a large number of microvessels in PMP tumor tissue through Doppler ultrasound and microangiography. Subsequent in vivo experiments showed that the survival time of bevacizumab-treated mice was significantly prolonged, blood vessels in tumor tissue gradually normalized, and the mean blood flow velocity slowed. In the same way, Andersson et al. [155] found that the expression levels of vascular endothelial markers (CD31 and CD105) and angiogenic factors VEGFA, fibroblast growth factor 2 (FGF2), and soluble Fms-like tyrosine1 (sFLT1) in PMP tumor tissue were significantly increased. In vivo studies have also shown that bevacizumab and aflibercept, an antiangiogenic drug targeting VEGFA, VEGFB, and placental growth factor (PlGF), both inhibit tumor growth to varying degrees, with the latter having a stronger effect. They suggest that PIGF and VEGFA are major targets for inhibiting PMP angiogenesis. At present, preliminary results regarding bevacizumab treatment have been reported for small samples of patients with recurrent PMP [46,135], but there is a lack of long-term prognostic evidence based on large samples. In addition, angiogenesis inhibition is mainly applied to palliative treatment in PMP, and its effect on preoperative neoadjuvant therapy or systemic treatment after CRS-HIPEC is unknown, which has certain research prospects.

### 5.3. Anti-Inflammatory Therapy

Mucin secretion is regulated by various mediators, including inflammatory cytokines [57,157]. Choudry et al. [158] investigated the effect of anti-inflammatory therapy on PMP through in vivo and in vitro experiments. The results showed that dexamethasone had dual inhibitory effects on the proliferation and mucin secretion of PMP tumor cells. In addition, Celebrex (COX-2 inhibitor) reduced MUC2 expression levels only in an inflammatory environment, and its effect in vivo was less significant than that of dexamethasone. This study suggests a new strategy for the treatment of PMP, but it is still necessary to further explore the mechanism of anti-inflammatory therapy in the future to provide sufficient preclinical research evidence for its widespread application.

### 5.4. Antibacterial Therapy

We previously described that bacteria (such as *Helicobacter pylori*) exist in PMP tumor tissues and may participate in the progression of PMP by influencing the secretion of MUC2 and inducing the EMT process [84,88,159]. Based on this, Merrell et al. [160] tracked the effect of perioperative anti-*H. pylori* therapy (lansoprazole, clarithromycin, and amoxicillin) on outcomes in patients with PMP (*n* = 17). The results in patients with low-grade PMP (*n* = 6) were as follows: five patients survived, and one patient was lost to follow-up. In those with high-grade PMP (*n* = 11), five patients died from complications of PMP, one died from other causes, and the remaining five patients survived. Although the prognosis is positive, especially for those with low-grade PMP, these benefits cannot be determined to be due to anti-*H. pylori* therapy. In addition, one of the commonly used basic drugs of HIPEC is mitomycin C, which, as an antibiotic, also has a certain killing effect on bacteria in the abdominal cavity [159]. Therefore, whether it is necessary to add antibiotic treatment during the perioperative period should be carefully considered. Moreover, more basic studies should be carried out to understand the role of bacteria in the progression of PMP to guide the use of antibiotics in PMP patients more scientifically.

### 5.5. Immunotoxin Therapy

Immunotoxins are bifunctional molecules composed of monoclonal antibodies and toxins that rely on monoclonal antibodies to bind to target cells and exert cytotoxic effects through toxins [161]. EpCAM is a type I transmembrane glycoprotein widely expressed in epithelial tumor cells such as the stomach, intestine, prostate, and lung [162]. In recent years, a new immunotoxin drug, MOC31PE, which is covalent between MOC31, a monoclonal antibody specific to EpCAM, and *Pseudomonas* exotoxin (PE) A, which is secreted by *Pseudomonas aeruginosa*, has become a hot topic in many studies. MOC31PE specifically binds to EpCAM-expressing tumor cells, inhibits protein synthesis, and induces cell apoptosis and *Phytophthora* cell death by releasing PE [163,164,165]. Currently, MOC31PE has been shown to have an encouraging prognosis in patients with peritoneal metastasis of colorectal cancer, with a 3-year OS of 72% and a median DFS of 13 months, and has demonstrated good safety and tolerability [166,167]. Flatmark et al. [165] found that EpCAM was also expressed in PMP samples. MOC31PE alone or combined with mitomycin C showed significant tumor growth inhibition, and the combined effect was more obvious. This suggests that MOC31PE combined with mitomycin C-based HIPEC may be a more effective treatment. Therefore, it is worth carrying out targeted clinical trials in PMP patients to further explore its actual efficacy and safety.

### 5.6. Targeted Hypoxia Therapy

Due to the rapid proliferation of tumor cells and the imbalance of oxygen supply, an anoxic environment is very common in most tumor tissues [168]. Hypoxia-inducible factor 1 (HIF-1) is a key transcription factor regulating oxygen homeostasis in the hypoxic microenvironment [169]. It is a heterodimer composed of two subunits, HIF-1α and HIF-1β. HIF-1β is structurally expressed, and HIF-1α is the main effector subunit. HIF-1α is degraded by ubiquitination in normal oxygen concentrations and is activated in low-oxygen environments to play an important regulatory role [170]. At present, hypoxia-targeted therapy targeting HIF-1α has achieved significant efficacy in preclinical models of various tumors [171,172]. In addition, HIF-1α has been found to play a catalytic role in the expression and secretion of mucin [173,174]. Valenzuela-Molina et al. [175] found that real-time oxygen microtension in soft and hard mucinous tumor tissues of PMP patients was significantly reduced during surgery, and HIF-1α protein expression levels were increased. These results indicate that a hypoxia environment exists in PMP. Dilly et al. [176] also found that the HIF-1α expression level was significantly increased in PMP tumor tissues, which could be associated with the MUC2 promoter (5′-ACGTGC-3′) interaction to regulate the expression level of MUC2. In addition, they found that HIF-1α inhibitors (YC-1 and BAY 87-2243) not only reduced the expression level of MUC2 but also effectively inhibited the progression of PMP in mouse xenotransplantation models. This study suggests that hypoxia in PMP regulates MUC2 expression by activating HIF-1α and that HIF-1α inhibitors may be an effective therapeutic strategy. 

### 5.7. Immune Checkpoint Inhibitor Therapy

Immune checkpoint inhibitor therapy is one of the most promising new tumor immunotherapies [177]. Immune checkpoints refer to a series of molecules that are expressed on immune cells and regulate the activation degree of the immune system, which is often compared to the “brake system” of immune cells to prevent the hyperactivation of the autoimmune system from causing damage to the body [178]. Programmed cell death protein 1 (PD-1) is a widely studied immune checkpoint. Its combination with programmed cell death ligand 1 (PD-L1) expressed on tumor cells can maintain the immune system in the “brake state”, which leads to the reduction in tumor cell-specific immunity and promotes tumor progression. In recent years, PD-1/PD-L1 inhibitors have shown significant and long-lasting inhibitory effects in a variety of tumors [179,180]. Relevant studies have shown that PD-1 is found in approximately 36% of PMP patients, and PD-L1 is found in approximately 16–18% [30,181]. Furthermore, the status of the MMR gene is related to the efficacy of anti-PD-1/anti-PD-L1 treatment, and mismatch repair deficiency (dMMR) patients treated with PD-1/PD-L1 inhibitors tend to have a better prognosis [182,183,184]. Yan et al. [53] conducted immunohistochemical tests on 155 PMP surgical specimens, and the incidence of dMMR was approximately 6.3%. For this subset of PMP patients, immune checkpoint inhibitor therapy may be a potential prognostic benefit.

### 5.8. Target Mitogen-Activated Protein Kinase (MAPK) Signaling Pathway Therapy

The MAPK signaling pathway has been reported in many studies to play an important role in mucin secretion [185,186]. The high mutation rate of KRAS and the high expression of MUC2 are typical characteristics of PMP. It is worth exploring whether the activation of the MAPK downstream signaling pathway mediated by KRAS mutation can play a role in regulating mucin secretion in PMP. Kuracha et al. [187] found that KRAS mutation activated the downstream PI3K/AKT and MEK/ERK signaling pathways, which were synergistically involved in maintaining MUC2 expression and tumor cell activity. A single application of a PI3K inhibitor (pictilisib) is prone to drug resistance, and in combination with an MEK inhibitor (cobimetinib), it can more effectively inhibit the expression of MUC2 and tumor growth. Dilly et al. [38] also confirmed that an MEK1/2 inhibitor (RDEA119) had a dual inhibitory effect on MUC2 expression and cell proliferation ability and that the use of RDEA119 in the peritoneal transplantation model of PMP mice could significantly reduce tumor load and prolong survival time. It may inhibit the expression levels of downstream nuclear factor kappa B (NF-kB) and activating protein 1 (AP1) through the MEK-ERK pathway; thus, the activity of the MUC2 promoter is decreased. In addition, the KRAS mutation rate is high in PMP patients, and Kras^G12D^ is one of the most common mutation subtypes [30]. Vázquez-Borrego et al. [188] used a small molecule inhibitor MRTX1133 that can target the Kras^G12D^ protein in a PMP mouse model. The results showed that it can effectively inhibit tumor growth by reducing the MAPK and PI3K/AKT/mTOR signaling pathways. This opens up a new treatment direction for PMP patients with Kras^G12D^ mutations and provides a strong theoretical basis for subsequent clinical trials.

### 5.9. Target Immunosuppressive Factors

Kusamura et al. [181] found the presence of immunosuppressive factors in PMP that are independent of KRAS and GNAS mutations, such as granulocyte macrophage colony-stimulating factor (GM-CSF) and A2A-adenosine receptor (A2AR). GM-CSF can further inhibit the anti-tumor ability of T lymphocytes by hindering the differentiation and maturation of dendritic cells [189]. A2AR can inhibit the activity of NK cells and CD8+ T cells through the c-AMP/PKA signaling pathway [190]. Relevant studies have shown that inhibiting GM-CSF and A2AR can effectively restore the activity of T cells, thereby alleviating immune suppression [191,192]. Therefore, targeting the inhibition of such immunosuppressive factors in PMP may be a potentially effective treatment modality.

## 6. Current Research Limitations

Although some progress has been made in research on PMP, there are still many limiting factors in clinical and basic research. (i) Low incidence and loss of disease source: In recent years, clinicians have gradually deepened their understanding of the disease, and the discovery rate has increased, but it is undeniable that the overall prevalence of PMP is still low. On this basis, sporadic cases around the world have not been further identified and evaluated at local experienced diagnosis and treatment centers after initial treatment at primary hospitals, resulting in a substantial loss of scarce disease sources. When clinical centers want to start clinical trials, it is difficult to recruit the target number of patients in a relatively short period of time, resulting in the slow progress of research and lengthening of the time to develop treatment strategies. (ii) Disordered pathologic classification: Before the four-classification system proposed by PSOGI was widely recognized in 2016, the pathological classification methods of PMP varied, and the application was chaotic. As a result, previous research data published by different international treatment centers cannot be used for reference or combined in subgroup studies based on pathological classification, which also has certain limitations in guiding the treatment selection and drug development of different pathological types of PMP. (iii) A lack of common cell lines: Due to the small number of PMP tumor cells and the large amount of mucus wrapped around them, it is difficult to achieve the cell concentration required for culture and eliminate the mucus around tumor cells during the construction of PMP primary cell lines. Currently, only four PMP cell lines have been reported to have been successfully cultured, N14 [193], N15 [193], NCC-PMP1-C1 [194], and NCC-PMP2-C1 [195]. However, these cell lines are not directly available to the majority of researchers. As a result, LS174T colon cancer cells, which can secrete mucin specifically, are used in almost all current or completed basic and preclinical studies on PMP. Some studies have been supplemented with the verification of a peritoneal xenotransplantation model of PMP tumor tissue in mice [147,165,176]. Due to the different tumor types, there may be some deviation in the mechanism studies and efficacy evaluation in the replacement cells. Therefore, the most urgent need at present is to cultivate more PMP cell lines and make them public to lay a solid foundation for the in-depth understanding of the molecular mechanism of this disease and the development of new therapeutic strategies.

## 7. Future Directions and Conclusions

In recent years, research on PMP has continued to advance, but there are still large gaps in clinical and basic research that need to be filled by further exploration in the future. On the clinical side, the effectiveness of HIPEC regimens and cycles, as well as neoadjuvant chemotherapy, postoperative intravenous chemotherapy, and their multimodal treatment, combined with targeted drugs, urgently need to be confirmed by larger-scale clinical trials, and standardized guidelines should be formed. For patients who cannot achieve CCRS, a unified standard needs to be established regarding what level of CRS can effectively reduce tumors, while minimizing surgical trauma and ensuring organ function. In addition, the PCI score is not well related to surgical difficulty and prognosis in PMP. For example, compared to patients with soft mucinous tumors with a PCI score of 39 and hard mucinous tumors with a PCI score of 12 but spread throughout the small intestine, the difficulty of surgery is significantly lower, the possibility of achieving CCRS is higher, and the prognosis is often better too. Therefore, it is necessary to establish a scoring system that is more suitable for the characteristics of PMP tumors. Consideration can be given to incorporating the hardness of mucinous tumor tissue into the scoring system and increasing the scoring value of difficult areas for surgery (left and right subphrenic, porta hepatis, small intestine, etc.).

In terms of basic research, the creation of mature and simple recognized cell lines is still the most urgent task to promote PMP research. Only on this basis can we explore the exact mechanism of the core issue of PMP mucinous tumor cells continuously producing mucin. In the context that this scientific problem cannot be solved at present, mucolytic treatment may be a research direction of great clinical significance. Recently, scholars have adopted chemical dissolution method (bromelain combined with N-acetylcysteine) and achieved positive results. Physical (ultrasound or microwave) and biological methods (bacteria [196]) may also be effective treatment strategies. Interestingly, where does the production of mucin come from in acellular mucin, a pathological type of PMP that does not have neoplastic epithelial cells in the peritoneal cavity? Whether the pathologists omitted the tumor cells in the face of a large number of PMP surgical specimens, whether the cell-free DNA of KRAS mutation played a role as suggested by García-Olmo et al. [197], or whether it was the colonization of *Pseudomonas* [86] or other reasons, the mechanism of this kind of tumor cell-independent mucin production merits further discussion.

In conclusion, PMP is a clinically rare disease characterized by the colonization of peritoneal mucinous tumor cells and the production of large amounts of mucus. The continuous secretion of mucus is a core issue in the development of the disease and the emergence of clinical symptoms, but due to the difficulty in constructing cell lines and the scarcity of cases, the exact mechanism is still unknown. The effectiveness of systemic chemotherapy is questionable, and CRS-HIPEC is still the preferred method for treating PMP. Further large-scale clinical trials and consensus on the selection of HIEPC regimens are needed. In addition, MTD-HIPEC may be a better option for patients who cannot achieve CCRS. However, it is undeniable that the single treatment of CRS/MTD-HIPEC has a high recurrence rate, and the era of multimodal treatment combined with targeted drugs or mucolytic therapy, which has great clinical translation prospects, needs to be opened. This strategy may revolutionize treatment for PMP patients and improve their prognosis and quality of life.

## Figures and Tables

**Table 1:** Common pathological grades of pseudomyxoma peritonei.

Ronnett (1995) [16]	PSOGI (2016) [1]	8th Edition AJCC Staging System (2017) [17]
NA	Acellular mucin (AM) Peritoneal lesions concentrated on or away from organ surfaces; composed of a large amount of mucin; without neoplastic epithelial cells	M1a
Disseminated peritoneal adenomucinosis (DPAM) Peritoneal lesions composed of abundant extracellular mucin and less focal mucinous epithelium; low cellular atypia; mitotic activity; with or without appendiceal mucinous adenoma	Low-grade mucinous carcinoma peritonei (LMCP)/Disseminated peritoneal adenomucinosis (DPAM) Peritoneal lesions show few low-grade epithelial cells (<20% of tumor volume); arranged in a single layer; mild cellular atypia; rare mitoses	M1b. G1 Well-differentiated
Peritoneal mucinous carcinomatosis (PMCA)/with intermediate feature (PMCA-I) Abundant epithelial cells in peritoneal lesions consistent with the architectural and cytological features of carcinoma; with or without primary mucinous adenocarcinoma/well-differentiated peritoneal mucinous carcinomatosis	High-grade mucinous carcinoma peritonei (HMCP)/peritoneal mucinous carcinomatosis (PMCA) Peritoneal lesions show abundant epithelial cells (>20% of tumor volume); high-grade histological features; infiltration of surrounding tissues; peripheral angiolymphatic and nerve invasion; cribriform growth Subclassification: - Well-differentiated (predominantly single-tubular glands; better cell polarity; obvious cellular atypia; invasive component) - Moderately differentiated (solid sheets of tumor cells with glandular structures; poor polarity) - Poorly differentiated (highly irregular to no adenoid structure; disappearance of cell polarity)	M1b. G2 or G3 Moderately or poorly differentiated
NA	High-grade mucinous carcinoma peritonei with signet ring cells (HMCP-S)/Peritoneal mucinous carcinomatosis with signet ring cells (PMCA-S) High-grade histology of peritoneal lesions with signet-ring cell component (signet-ring cells ≥10%)	M1b. G3 Poorly differentiated; PMCA-S

PSOGI, Peritoneal Surface Oncology Group International; AJCC, American Joint Committee on Cancer; NA, not applicable.

**Table 2:** PSOGI expert consensus on contraindications to CCRS/HIPEC.

Contraindication	Description	PSOGI Expert Consensus Rate
Absolute	Retraction due to mesenteric involvement	64.3%
	Extensive involvement of the small bowel serosa, unable to preserve 1.5–2 m of small bowel without tumor invasion	58.9%
Relative	PCI > 20 with aggressive histology (e.g., mucinous adenocarcinoma with signet ring cells, goblet cell carcinoid, and high-grade PMP with signet ring cells)	87.5%
	Massive involvement of the liver hilum	87.5%
	Age > 75 years old	85.7%
	Extensive Infiltration of the pancreatic surface	82.1%
	Requires complete gastrectomy	80.4%
	Ureteral obstruction	64.3%

PSOGI, Peritoneal Surface Oncology Group International; CCRS, complete cytoreduction surgery (cytoreductive to CC0 or CC1); HIPEC, hyperthermic intraperitoneal chemotherapy; PCI, peritoneal cancer index.

**Table 3:** Commonly used HIPEC regimens for pseudomyxoma peritonei.

HIPEC Regimens (PSOGI Expert Consensus Rate)	Dose	Time	Intraperitoneal Component	Intravenous Component
Dutch High-Dose Mitomycin C Regimen: “Triple Dosing Regimen” (42.9%) [113]	35 mg/m^2^	90 min	Mitomycin C was added to 1.5% peritoneal dialysis solution at an initial dose of 17.5 mg/m^2^, followed by 8.8 mg/m^2^ after 30 min and 8.8 mg/m^2^ after 60 min	NA
Glehen Medium-Dose Oxaliplatin Regimen (28.6%) [110]	360 mg/m^2^	30 min	Add oxaliplatin to 2 L/m^2^ 5% dextrose solution and maintain intraperitoneal chemotherapy for 30 min	1 h before intraperitoneal chemotherapy, 5-fluorouracil 400 mg/m^2^ and leucovorin 20 mg/m^2^ were separately added to 250 mL of normal saline for rapid intravenous infusion
American Society of Peritoneal Surface Malignancy Low-Dose Mitomycin C Regimen: “Concentration-Based Regimen” (14.3%) [111]	40 mg/3L	90 min	Add mitomycin C to 1.5% peritoneal dialysis solution, the initial dose is 30 mg/3 L, and then add 10 mg after 60 min	NA
PMI Basingstoke IP Chemotherapy Regimen: “Body Surface Area-Based” (10.7%) [92]	10 mg/m^2^	60 min	Add mitomycin C to 0.9% sodium chloride solution at 42 °C. Reduce the dose by 33% for obesity (BMI > 40), severe abdominal distension, and severe chemotherapy in the past 3 months	NA
Elias High-Dose Oxaliplatin Regimen (8.9%) [109]	460 mg/m^2^	30 min	Add oxaliplatin to 2 L/m^2^ 5% dextrose solution and maintain intraperitoneal chemotherapy for 30 min	1 h before intraperitoneal chemotherapy, 5-fluorouracil 400 mg/m^2^ and leucovorin 20 mg/m^2^ were separately added to 250 mL of normal saline for rapid intravenous infusion
Wake Forest University Oxaliplatin Regimen (1.8%) [110]	200 mg/m^2^	120 min	Add oxaliplatin to 5% dextrose solution and maintain intraperitoneal chemotherapy for 120 min	NA
Sugarbaker Regimen (1.8%) [114]	15 mg/m^2^	90 min	Add 15 mg/m^2^ of mitomycin C and doxorubicin to 2 L 1.5% dextrose peritoneal dialysis solution and maintain intraperitoneal chemotherapy for 90 min	At the same time of intraperitoneal chemotherapy, 5-fluorouracil 400 mg/m^2^ and leucovorin 20 mg/m^2^ were separately added to 250 mL of normal saline for rapid intravenous infusion

PSOGI, Peritoneal Surface Oncology Group International; HIPEC, hyperthermic intraperitoneal chemotherapy; NA, not applicable.

## Data Availability

No new data were created or analyzed in this study.

## Abbreviations

A2AR	A2A-adenosine receptor
AP1	Activating protein 1
APC	APC regulator of WNT signaling pathway
ATF1	Activating transcription factor 1
cAMP	Cyclic adenosine monophosphate
CC	Completeness of the cytoreduction
CCRS	Complete cytoreduction surgery
CDH1/2	Cadherin 1/2
CLDN3/4	Claudin 3/4
CREB	cAMP responsive element binding protein
CRS	Cytoreductive surgery
DCR	Disease control rate
DFS	Disease-free survival
dMMR	Mismatch repair deficiency
DPAM	Disseminated peritoneal adenomucinosis
EMT	Epithelial–mesenchymal transition
EpCAM	Epithelial cell adhesion molecule
EPIC	Early postoperative intraperitoneal chemotherapy
ESRP1	Epithelial splicing regulatory protein 1
FGF2	Fibroblast growth factor 2
FN1	Fibronectin 1
GM-CSF	Granulocyte macrophage colony-stimulating factor
GNAS	Guanine nucleotide-binding protein alpha subunit
HIF-1	Hypoxia-inducible factor 1
HIPEC	Hyperthermic intraperitoneal chemotherapy
HITHOC	Hyperthermic intrathoracic chemotherapy
IL-1β/9/13	Interleukin 1 beta/9/13
KLRF1/G1	Killer cell lectin-like receptor F1/G1
KRAS	Kirsten rat sarcoma viral oncogene homologue
MMR	Mismatch repair
MAPK	Mitogen-activated protein kinase
MTD	Maximum tumor debulking
MUC	Mucin
NAC	N-acetyl cysteine
NF-kB	Nuclear factor kappa B
OS	Overall survival
PCI	Peritoneal cancer index
PD-1	Programmed cell death protein 1
PD-L1	Programmed cell death ligand 1
PFS	Progression-free survival
PIGF	Placental growth factor
PIK3CA	Phosphatidylinositol-4,5-bisphosphate 3-kinase catalytic subunit alpha
PIPAC	Pressurized intraperitoneal aerosol chemotherapy
PKA	Protein kinase A
PMCA	Peritoneal mucinous carcinomatosis
PMCA-S	Peritoneal mucinous carcinomatosis with signet ring cells
PMP	Pseudomyxoma peritonei
PSOGI	Peritoneal Surface Oncology Group International
RAR-α	Retinoic acid receptor alpha
sFLT1	Soluble Fms-like tyrosine1
SMAD4	SMAD family member 4
SPINK1	Serine peptidase inhibitor Kazal type 1
STAT3	Signal transducer and activator of transcription 3
TNF-α	Tumor necrosis factor alpha
TRA	T-cell receptor alpha locus
TRBC1	T-cell receptor beta constant 1
VEGFA/B	Vascular endothelial growth factor A/B
VIM	Vimentin
